# Possible mechanism of benign prostatic hyperplasia induced by androgen–estrogen ratios in castrated rats

**DOI:** 10.4103/0253-7613.70397

**Published:** 2010-10

**Authors:** Liu Xiang-Yun, Xu Ying-Wen, Xie Chen-Jing, Wang Jiu-Jiu, Pan Qi, Gui Bo, Sun Zu-Yue

**Affiliations:** Department of Pharmacology and Toxicology, Shanghai Institute of Planned Parenthood Research, National Evaluation Centre for the Toxicology of Fertility Regulation Drugs, Shanghai 200032, China

**Keywords:** Area of prostate glandular cavity, height of prostate epithelia, organ quotient

## Abstract

**Objectives:**

To explore the role of androgen–estrogen balance in benign prostatic hyperplasia (BPH) induced by varying doses of estradiol/testosterone propionate (E_2_/TP) in castrated rats.

**Materials and Methods:**

A total of 222 rats were divided into 37 groups at random, including 35 groups of different E_2_/TP, one control, and one castrated group. All 37 groups except the control group were castrated, for eliminating endogenesis of testosterone in rats. The treated groups were administered testosterone propionate (TP; at the dosages of 0.15, 0.74, 3.7, 18.5, and 92.6 mg/kg), combined with estradiol (E_2_; at the dosage of 0, 0.4, 2, 10, 50, 250, and 1250 µg/kg) diluted in vegetable oil for 30 days, respectively, whereas the control groups received only vegetable oil. All prostate specimens were removed under anesthesia, then fixed and embedded in paraffin, for measuring the organ quotient, volume, area of prostate glandular cavity, and the height of prostate epithelia.

**Results:**

When the dosages of TP were 0.15, 3.7, 18.5, and 92.6 mg/kg, the degree of prostatic hyperplasia had no obvious dose–effect relationship with E_2_. When TP was 0.74 mg/kg, with the increase of the dosage of E_2_, the volume and quotient of prostate were increasing. However, when the dosage of E_2_exceeded 50 µg/kg, E_2_/TP was 5/74, the prostatic volume did not increase obviously.

**Conclusion:**

The proper levels of E2/TP play an important role in the pathogenesis of BPH. In rats, the balance point of E_2_/TP is 5/74.

## Introduction

Benign prostatic hyperplasia (BPH) is a common disease of men over 50, and its incidence goes up with advancing age. Statistics shows that BPH is hardly found in men less than 30 years old,[1] but in 88% of autopsies BPH were found in men aged above 80,[2] with compatible symptomatology reported in nearly 50% of men aged above 50 in the general population. The phenomenon maybe correlated with changes of sex hormone in serum of elderly population. One clinical study reported that there were low free testosterone concentrations with relative rise in serum estradiol levels in patients of BPH.[3] Testosterone level declines with age, but serum estrogen level remains unaltered, so estrogen may be involved in the development of BPH.[4]

BPH is histologically complex, involving glandular and stromal hyperplasia, fibrosis, and prostatitis.[5] The etiology of BPH is still poorly understood. It is thought to be related to the combination of aging and endocrine dysregulation. It is well documented that androgens are the primary factor for prostate disease,[6,7] but the mechanism is still unclear.[8] While the prostate is considered the prototype androgen-dependent gland, there is rising evidence that estrogen is necessary to maintain the natural function of prostate.[9,10] In addition, estrogens also play an important role in growth and differentiation of prostate gland.[11] We have tried to find a balance point of estradiol/testosterone propionate (E_2_/TP) in development of BPH. The results may provide further insight into the role of E_2_/TP in development of BPH.

## Materials and Methods

### Animal

All experiments were performed in Shanghai. Male Sprague-Dawley rats (120 ± 10 g) were obtained from the Shanghai SIPPR-BK (Shanghai Institute of Planned Parenthood Research—BK Laboratory Animal Limited Company, Shanghai, China). The animals were weighed and kept under the same conditions with free access to water and food. A total of 222 rats were divided in 37 groups with six animals in each group at random, including 35 groups of different E_2_/TP, one control group and another group of castrated animals which also served as sham control. Except the rats of control group, all rats were castrated under anesthesia with ketamine, for eliminating endogenesis of testosterone. The experiments were started in the first week after castration.

### Study procedures

As testosterone propionate (TP) (3.7 mg/kg) is shown to result in rat BPH,[12] the treated groups were administered (*s.c.*) daily with different dosages of TP (five doses of 0.15, 0.74, 3.7, 18.5, and 92.6 mg/kg) combined with different dosages of estradiol (E_2_) (seven doses of 0, 0.4, 2, 10, 50, 250, and 1250 µg/kg) diluted in vegetable oil for 30 days, respectively, whereas the control groups received only vegetable oil. All prostate specimens were removed under anesthesia, then fixed and embedded in paraffin. The paraffin-blocked section was consecutively cut at 5-*µ*m thickness for hematoxylin–eosin (H&E) and immunohistochemical staining. After the prostate quotient (the weight of prostate/the weight of rats) and the volume were determined, the area of 200 glandular cavity and 200 height of prostatic epithelia of prostate in each tissues slide were measured by image analysis software after being shot by camera under light microscope. Furthermore, AR-labeled cells were detected using immunohistochemical staining as described. After deparaffination and rehydration of sections, slides were placed in sodium citrate solution (0.01 M, pH 6.0) and heated to 96–100°C for 25 min. After cooling, sections were put into 5% BSA for 20 min. Then, sections were incubated for 2 h with primary anti-AR mouse monoclonal antibody (Boster Biotechnology Co. Ltd. Wuhai, China) diluted 1:100 in TBS, lastly, covered with cover slips. AR-labeled cells were observed under a light microscope.

### Statistics

Data were expressed as mean ±SD. One-way ANOVA and *P* values were used to evaluate significant differences between the groups. Image-Pro Plus 6.0 was used to analysis image data.

## Results

### Changes in prostate after treatment

#### Effect of different dosages of E2 with TP (0.15 mg/kg)

**Figure 1:**
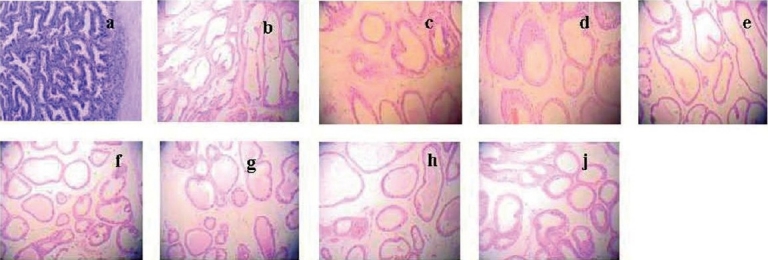
Effect of E_2_ on prostate pathology when TP was 0.15 mg/kg/rat. Compared with the control (a) and castrated control (b) group, the area of prostate glandular cavity and the height of prostate epithelia had no obvious change. 7 groups which were all administered TP 0.15 mg/kg, and E_2_ were 0 (c), 0.4 (d), 2.0 (e), 10 (f), 50 (g), 250 (h) and 1250µg/kg (j). Magnification ×100.

There were seven groups, in which all rats were administered with TP of 0.15 mg/kg, with different dosages of E_2_ (0, 0.4, 2.0, 10, 50, 250, and 1250 µg/kg), respectively. Compared with the castrated control group, the organ quotient, volume and area of prostate glandular cavity, and the height of prostate epithelia were found to be statistically comparable (*P* > 0.05) [Table 1 and Figure 1]. The prostates of the animals from all the treatment groups were found to be regressed, and the organ quotient and the volume of prostate were significantly less (*P* < 0.05) [Table 1], compared to the control group. There were no significant differences in the area of prostate glandular cavity and height of prostate epithelia (*P* > 0.05) [Table 1 and Figure 1].

**Table 1:** Effect of E_2_ on prostate when TP was 0.15 mg/kg (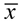 ± SD)

*E_2_ (*µ*g/kg)*	*N*	*weight (g)*	*Organ quotient (/100)*	*Volume (mL)*	*Prostate epithelia height (*µ*m)*	*Glandular cavity area (*µ*m^2^)*
Control	6	309.25 ± 10.72	1.50 ± 0.06	0.12 ± 0.05	16.95 ± 5.98	23956 ± 8978
Castrated	6	297.25 ± 15.73	0.35 ± 0.02	0.06 ± 0.03	15.77 ± 3.56	23606 ± 17981
0	6	334.50 ± 31.59	0.81 ± 0.31*	0.10 ± 0*	13.81 ± 8.74	20917 ± 19095
0.4	6	327.00 ± 30.56	0.65 ± 0.09*	0.06 ± 0.02*	12.70 ± 5.12	20361 ± 14851
2.0	6	306.00 ± 24.05	1.02 ± 0.36*	0.10 ± 0.04	11.90 ± 5.25	27854 ± 2689
10	6	274.25 ± 7.63	1.48 ± 0.24*	0.14 ± 0.05*	13.56 ± 3.97	11368 ± 10058
50	6	274.50 ± 17.48	1.28 ± 0.43*	0.10 ± 0.04*	14.21 ± 7.04	10987 ± 9380
250	6	236.50 ± 26.13	1.28 ± 0.16*	0.08 ± 0.02*	14.21 ± 3.85	10594 ± 10274
1250	6	202.25 ± 16.68	1.34 ± 0.48*	0.05 ± 0.03*	11.61 ± 4.22	17333 ± 15449

Note: Compared with control group,

* *P* < 0.05.

#### Effect of different dosages of E_2_ with TP (0.74 mg/kg)

**Figure 2:**
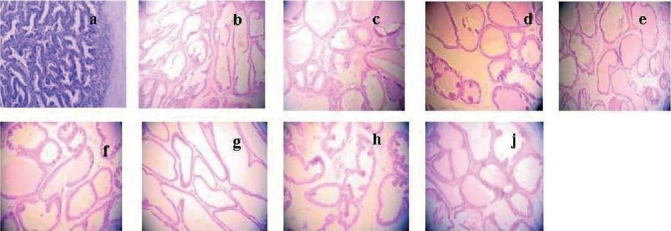
Effect of E_2_ on prostate pathology when TP was 0.74 mg/kg/rat. Control group is picture (a) and castrated control group is picture (b). And the other 7 groups which were all administered TP 0.74 mg/kg/rat, and E_2_ were 0 (c), 0.4 (d), 2.0 (e), 10 (f), 50 (g), 250 (h) and 1250µg/kg (j). Compared with the control (a) and castrated control (b) group, the area of prostate glandular cavity of the last thee groups (g, h, and j) increased significantly. Magnification ×100.

There were seven groups, in which all rats were administered with TP of 0.74 mg/kg, with different dosages of E_2_ (0, 0.4, 2.0, 10, 50, 250, and 1250 µg/kg), respectively. Compared with the group which received TP alone, the prostate organ quotient and the volume of the other groups (E_2_ ≥ 0.4 µg/kg) were enlarged significantly (*P* < 0.05) [Table 2]. In addition, individual groups compared with each other. There was no statistically significant difference (*P* > 0.05) among the middle three groups (E_2_ doses 0.4, 2.0, and 10 µg/kg) and the last three groups (E_2_ doses 50, 250, and 1250 µg/kg), but compared with the middle three groups, each of the last three groups was enlarged significantly (*P* < 0.05) [Table 2]. The height of prostate epithelia of the two groups (E_2_ doses 250, 1250 µg/kg) was higher than the control groups, but the differences were insignificant (*P* > 0.05). Compared with the control group, the area of prostate glandular cavity of the last three groups was observed to be increased and the increase was statistically significant (*P* < 0.05) [Table 2 and Figure 2].

**Table 2:** Effect of E_2_ on prostate when TP was 0.74 mg/kg/rat (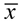 ± SD)

*E_2_ (*µ*g/kg)*	*N*	*weight (g)*	*Organ quotient (/100)*	*Volume (mL)*	*Prostate epithelia height (*µ*m)*	*Glandular cavity area (*µ*m^2^)*
Control	6	309.25 ± 10.72	1.50 ± 0.06	0.12 ± 0.05	16.95 ± 5.98	23956 ± 8978
Castrated	6	297.25 ± 15.73	0.35 ± 0.02	0.06 ± 0.03	15.77 ± 3.56	23606 ± 17981
0	6	185.00 ± 14.58	1.71 ± 0.49	0.17 ± 0.04	13.66 ± 6.47	11360 ± 15059
0.4	6	214.25 ± 15.26	2.96 ± 0.38*	0.27 ± 0.05*	16.44 ± 6.31	22834 ± 2239
2.0	6	206.25 ± 19.17	3.05 ± 0.66*	0.29 ± 0.11*	11.93 ± 4.60	19935 ± 18697
10	6	185.00 ± 94.19	2.91 ± 0.27*	0.27 ± 0.09*	15.73 ± 7.49	21804 ± 24157
50	6	218 ± 14.65	3.5 ± 1.02*	0.84 ± 0.23*	15.73 ± 7.49	24547 ± 29240*
250	6	192.75 ± 7.27	3.8 ± 0.91*	0.61 ± 0.12*	19.84 ± 6.27	26842 ± 28461*
1250	6	183.50 ± 10.84	3.6 ± 1.05*	0.75 ± 0.21*	24.94 ± 21.97	23551 ± 19144*

Note: Compared with the group (E_2_ = 0 µg/kg),

* *P* < 0.005.

#### Effect of different dosages of E_2_ with TP (3.7 mg/kg)

**Figure 3:**
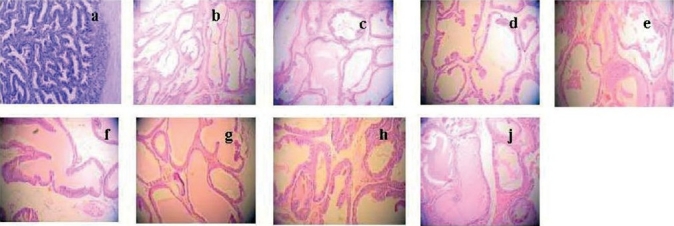
Effect of E_2_ on prostate pathology when TP was 3.7 mg/kg/rat. Control group is picture (a) and castrated control group is picture (b). And the other 7 groups which were all administered TP 3.7 mg/kg/rat, and E_2_ were 0 (c), 0.4 (d), 2.0 (e),10 (f), 50 (g), 250 (h), 1250µg/kg (j). Compared with control group (a), the area of prostate glandular cavity were significant differences. Magnification ×100.

There were seven groups which were all administered with TP of 3.7 mg/kg, with different dosages of E_2_ (0, 0.4, 2.0, 10, 50, 250, and 1250 µg/kg), respectively. Compared with the control groups, the organ quotient, the volume, and the area of prostate glandular cavity were all increased significantly (*P* < 0.01) [Table 3 and Figure 3]. The prostate epithelia appeared high stylolitic with increase in glandular cavity, and the area of prostate glandular cavity was large.

**Table 3:** Effect of E_2_ on prostate when TP was 3.7 mg/kg/rat (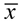 ± SD)

*E_2_ (*µ*g/kg)*	*N*	*weight (g)*	*Organ quotient (/100)*	*Volume (mL)*	*Prostate epithelia height (*µ*m)*	*Glandular cavity area (*µ*m^2^)*
Control	6	309.25 ± 10.72	1.50 ± 0.06	0.12 ± 0.05	16.95 ± 5.98	23956 ± 8978
Castrated	6	297.25 ± 15.73	0.35 ± 0.02	0.06 ± 0.03	15.77 ± 3.56	23606 ± 17981
0	6	297.25 ± 15.73	4.66 ± 0.30**	1.23 ± 0.13**	19.16 ± 6.26	38293 ± 30062**
0.4	6	192.75 ± 18.42	5.81 ± 0.30**	1.35 ± 0.23**	18.24 ± 8.94	35726 ± 35316**
2.0	6	206.25 ± 15.11	4.67 ± 0.74**	1.40 ± 0.23**	20.18 ± 9.54	48435 ± 57070c**
10	6	204.00 ± 8.04	5.53 ± 0.15**	1.25 ± 0.07**	23.12 ± 8.28	40940 ± 32754**
50	6	211.00 ± 6.16	5.33 ± 0.39**	1.13 ± 0.16**	19.56 ± 5.79	41500 ± 38459**
250	6	210.50 ± 16.36	5.44 ± 1.58**	0.97 ± 0.27**	25.31 ± 8.01	55851 ± 65307**
1250	6	202.5 ± 16.30	6.16 ± 1.51**	1.31 ± 0.42**	19.79 ± 6.73	34370 ± 28379**

Note: Compared with control groups,

** *P* < 0.01.

#### Effect of different dosages of E_2_ with TP (18.5 mg/kg)

**Figure 4:**
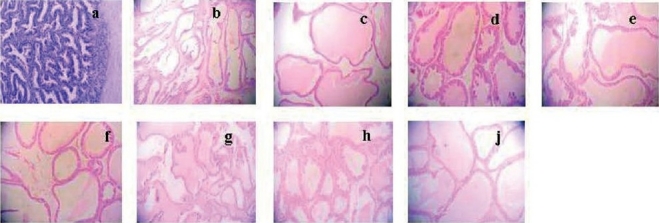
Effect of E_2_ on prostate pathology when TP was 18.5 mg/kg/rat. Control group is picture (a) and castrated control group is picture (b). And the other 7 groups which were all administered TP 18.5 mg/kg/rat, and E_2_ were 0 (c), 0.4 (d), 2.0 (e), 10 (f), 50 (g), 250 (h) and 1250 µg/kg (j). Compared with the control group (a), the area of prostate glandular cavity were all significant differences. Magnification ×100.

There were seven groups which were all administered with TP of 18.5 mg/kg, with different dosages of E_2_ (0, 0.4, 2.0, 10, 50, 250, and 1250 µg/kg), respectively. Compared with the control groups, the organ quotient, the volume, and the area of prostate glandular cavity were all significantly different (*P* < 0.01) [Table 4 and Figure 4]. The prostate epithelia appeared high stylolitic with increased glandular cavity, and the area of prostate glandular cavity was large.

**Table 4:** Effect of E_2_ on prostate when androgen was 18.5 mg/kg/rat (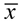 ± SD)

*E_2_ (*µ*g/kg)*	*n*	*weight (g)*	*Organ quotient (/100)*	*Volume (mL)*	*Prostate epithelia height (*µ*m)*	*Glandular cavity area (*µ*m^2^)*
Control	6	309.25 ± 10.72	1.50 ± 0.06	0.12 ± 0.05	16.95 ± 5.98	23956 ± 8978
Castrated	6	297.25 ± 15.73	0.35 ± 0.02	0.06 ± 0.03	15.77 ± 3.56	23606 ± 17981
0	6	208.50 ± 2.89	6.25 ± 0.45**	1.75 ± 0.18**	31.72 ± 160	37307 ± 23483**
0.4	6	206.50 ± 7.89	5.73 ± 0.87**	1.55 ± 0.24**	26.13 ± 7.97	33329 ± 25383**
2.0	6	189.50 ± 4.43	4.94 ± 0.47**	1.26 ± 0.19**	23.66 ± 7.60	44317 ± 26342**
10	6	216.00 ± 13.74	5.60 ± 1.20**	1.61 ± 0.29**	27.01 ± 9.24	42314 ± 31836**
50	6	211.25 ± 11.25	7.03 ± 0.98**	1.70 ± 0.30**	18.15 ± 6.32	38829 ± 31514**
250	6	194.00 ± 13.74	6.77 ± 0.4**	1.56 ± 0.09**	23.30 ± 9.41	38232 ± 26268**
1250	6	192.00 ± 15.30	5.98 ± 0.66**	1.37 ± 0.13**	17.47 ± 6.45	34358 ± 25225**

Note: Compared with control groups,

** *P* < 0.01.

#### Effect of different dosages of E_2_ with TP (92.6 mg/kg)

**Figure 5:**
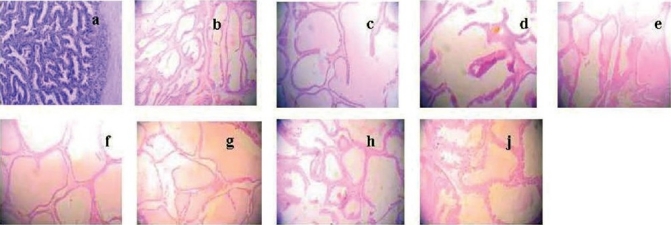
Effect of E_2_ on prostate pathology when TP was 92.6 mg/kg/rat. Control group is picture (a) and castrated control group is picture (b). And the other 7 groups which were all administered TP 92.6 mg/kg/rat, and E_2_ were 0 (c), 0.4 (d), 2.0 (e), 10 (f), 50 (g), 250 (h) and 1250µg/kg (j). Compared with the castrated control group (a), the area of prostate glandular cavity were all significant differences. Magnification ×100.

There were seven groups which were all administered with TP of 92.6 mg/kg, with different dosages of E_2_ (0, 0.4, 2.0, 10, 50, 250, and 1250 µg/kg), respectively. Compared with the control group, the organ quotient, the volume, the area of prostate glandular cavity were all significantly different (*P* < 0.01) [Table 5 and Figure 5]. The prostate epithelia appeared high stylolitic with increased glandular cavity, and the area of prostate glandular cavity was large. However, when the dosages of TP exceed 3.7 mg/kg, E_2_ had little effect on prostate as compared to other groups (*P* < 0.05).

**Table 5:** Effect of E_2_ on prostate when TP was 92.6 mg/kg/rat (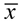 ± SD)

*E_2_ (*µ*g/kg)*	*n*	*weight (g)*	*Organ quotient (/100)*	*Volume (mL)*	*Prostate epithelia height (*µ*m)*	*Glandular cavity area (*µ*m^2^)*
Control	6	309.25 ± 10.72	1.50 ± 0.06	0.12 ± 0.05	16.95 ± 5.98	23956 ± 8978
Castrated	6	297.25 ± 15.73	0.35 ± 0.02	0.06 ± 0.03	15.77 ± 3.56	23606 ± 17981
0	6	183.25 ± 10.53	6.48 ± 1.34**	1.68 ± 0.26**	17.97 ± 7.14	45362 ± 23746**
0.4	6	191.75 ± 15.76	6.64 ± 1.13**	1.52 ± 0.24**	20.27 ± 7.47	49886 ± 47606**
2.0	6	207.75 ± 4.99	7.00 ± 0.14**	1.73 ± 0.32**	15.26 ± 6.3	56084 ± 60502**
10	6	198.25 ± 16.15	6.31 ± 0.84**	1.72 ± 0.23**	21.76 ± 7.59	45691 ± 37235**
50	6	189.25 ± 8.54	7.26 ± 1.08**	1.89 ± 0.16**	21.21 ± 9.74	39398 ± 29637**
250	6	203.5 ± 21.50	7.31 ± 0.56**	2.07 ± 0.37**	24.35 ± 74.14	35666 ± 35746**
1250	6	198.51 ± 21.69	6.54 ± 1.91**	1.59 ± 0.33**	25.30 ± 9.49	35865 ± 28903**

Note: Compared with control groups,

** *P* < 0.001.

### Androgen receptor-labeled cell assay

#### Effect of E2 on androgen receptor (AR) of prostate with TP (0.15 mg/kg)

There were seven groups which were all administered with TP of 0.15 mg/kg, with different dosages of E_2_ (0, 0.4, 2.0, 10, 50, 250, and 1250 µg/kg), respectively. A large number of cells were observed in the areas of prostate epithelial cells and stroma, but positive cells were hardly seen in all groups.

#### Effect of E_2_ on androgen receptor (AR) of prostate with TP (0.74 mg/kg)

There were seven groups which were all administered with TP of 0.74 mg/kg, with different dosages of E_2_ (0, 0.4, 2.0, 10, 50, 250, and 1250 µg/kg), respectively. A few of AR-labeled cells were observed in the last three groups (E_2_ were 50, 250, and 1250 µg/kg), whereas positive cells were hardly seen in the control and the other treatment groups.

#### Effect of E_2_ on androgen receptor (AR) of prostate with TP (3.7 mg/kg)

There were seven groups which were all administered with TP of 3.7 mg/kg, with different dosages of E_2_ (0, 0.4, 2.0, 10, 50, 250, and 1250 µg/kg), respectively. AR-labeled cells appeared in all treatment groups, and especially, a number of AR-labeled positive cells were observed in the last three groups (E_2_ were 50, 250, and 1250 µg/kg).

#### Effect of E_2_ on androgen receptor (AR) of prostate with TP (18.5 mg/kg)

There were seven groups which were all administered with TP of 18.5 mg/kg, with different dosages of E_2_ (0, 0.4, 2.0, 10, 50, 250, and 1250 µg/kg), respectively. AR-labeled cells appeared in all treatment groups, but there were insignificant differences in them.

#### Effect of E_2_ on androgen receptor (AR) of prostate with TP (92.6 mg/kg)

There were seven groups which were all administered with TP of 92.6 mg/kg, with different dosages of E_2_ (0, 0.4, 2.0, 10, 50, 250, and 1250 µg/kg), respectively. AR-labeled cells appeared in all treatment groups to similar extent, AR-labeled cells were not found and compared statistically.

## Discussion

Androgens play an obligatory role in the embryonic development and function of prostate gland in adults. The essential role of androgens in prostatic development is clearly evident in genetic XY males with congenital abnormality in AR function or deficiency in 5-α-reductase, since in these individuals the prostate is either absent or incompletely developed.[13] The secretory epithelial cells express the AR, and they require continuous androgenic stimulation for survival and functional integrity. When the androgen level drops below a threshold, as is the case after surgical or chemical castration, the secretory cells undergo apoptosis, causing glandular involution.[14] In the study, when androgen is 0.15 mg/kg, even if the highest dosage (1250 µg/kg) of E_2_ were administered, there were no obvious changes in the organ quotient, volume, area of prostate glandular cavity, and height of prostate epithelia. In addition, the AR-labeled cells were hardly seen through immunohistochemical examination. When the dosage of TP was 0.74 mg/kg, with the increasing of the dosage of E_2_, the volume and quotient of prostate increased. When the dosages of E_2_ were 50, 250, and 1250 µg/kg in TP—0.74 mg/kg group, the area of prostate glandular cavity increased and a few little AR-labeled cells appeared. The results proved that when the TP dose was below 0.15 mg/kg, the prostate gland showed atrophy, whereas when TP dose was 0.74 mg/kg but the prostate gland was found to be hyperplastic when TP dose was 0.74 mg/kg. When TP was over 3.7 mg/kg, the organ quotient, volume, area of prostate glandular cavity showed further increase which was obvious and AR express markedly, which support the fact that androgen is a crucial hormone for prostate development.[15]

It was observed that when TP was 0.74 mg/kg combined with E_2_ (0.4 µg/kg), i.e., E_2_/TP was 2/3700, the change in morphology of prostate was less; however when E_2_ is 50 µg/kg, i.e., E_2_/TP of 5/74, there was obvious change in prostate gland structure. Estrogen does not always cause antiandrogen effects, but under specific conditions, it may be of benefit to induce prostatic hyperplasia.[16–18] Many studies reported that estrogens affect prostatic hyperplasia. This neonatal exposure to estradiol resulted in a permanent reduction in prostatic growth and activational response to androgens during adulthood, an effect mediated in part through a permanent reduction in AR expression.[19] Exposure to estradiol results in neonatal results in promoting prostate hyperplasia during adulthood. The effects of estrogens on prostate were found to be complicated.[19]

Therefore, we attempted to study the effect of E_2_/TP on prostate. Serum level of estrogen–androgen is 1/150 in adulthood. The incidence of BPH is related to age. With increasing age, serum level of estrogen–androgen is 1/120–1/80 in elderly, whereas it can reach 1/8 in prostate.[20] BPH could be induced by the change of E_2_/TP. Mark reported that dihydrotestosterone (DHT) plus E_2_ treatment in animals increased the prostatic activity of 4-hydroxy estradiol synthase, whereas either E_2_ or DHT treatment alone did not change this activity.[21] Our results show that in rats, balance point of E_2_/TP is 5/74. The proper E_2_/TP ratio plays an important role in the pathogenesis of BPH. If the optimum ratio is not maintained, it can lead to BPH. This knowledge of optimizing E_2_/TP in humans may help to prevent or cure BPH in future.
